# Targeted tumor therapy by *Rubia tinctorum* L.: analytical characterization of hydroxyanthraquinones and investigation of their selective cytotoxic, adhesion and migration modulator effects on melanoma cell lines (A2058 and HT168-M1)

**DOI:** 10.1186/s12935-015-0271-4

**Published:** 2015-12-18

**Authors:** Eszter Lajkó, Péter Bányai, Zsófia Zámbó, László Kursinszki, Éva Szőke, László Kőhidai

**Affiliations:** Department of Genetics, Cell- and Immunobiology, Semmelweis University, Nagyvárad tér 4, Budapest, 1089 Hungary; Department of Pharmacognosy, Semmelweis University, Üllői út 26, Budapest, 1085 Hungary

**Keywords:** *Rubia tinctorum* L., Hydroxyanthraquinone, Purpurin, Melanoma, Targeted therapy, HPLC–MS/MS, Cell adhesion, Migration, Impedimetry, Holographic microscope

## Abstract

**Background:**

Alizarin and purpurin are di- and trihydroxyanthraquinones derived from *Rubia tinctorum* L. Previous pharmacological studies have demonstrated that they exhibit certain degree of selective inhibitory effects towards cancer cells suggesting their application as a targeted drug for cancer. Our present work was aimed to investigate the suitability of hydroxyanthraquinones of *Rubia tinctorum* L. for targeted tumor therapy. The effects of alizarin, purpurin and an aqueous extract from transformed hairy root culture of *Rubia tinctorum* L. were examined on (1) cell proliferation, (2) apoptosis, (3) cell adhesion/morphology and (4) migration (chemotaxis, chemokinesis) of human melanoma cell lines (A2058, HT168-M1) and human fibroblast cells (MRC-5), as well as (5) the aqueous extract was analytically characterized.

**Methods:**

The aqueous extract was prepared from *R. tinctorum* hairy root culture and qualitatively analyzed by HPLC and ESI–MS methods. The cell growth inhibitory activity of anthraquinones was evaluated by MTT-assay and by flow cytometry. The effect of anthraquinones on cell adhesion was measured by an impedance based technique, the xCELLigence SP. For the chemotaxis assay NeuroProbe^®^ chamber was used. Computer based holographic microscopy was applied to analyze chemokinetic responses as well as morphometry. Statistical significance was determined by the one-way ANOVA test.

**Results:**

In the aqueous extract, munjistin (M_r_ = 284, t_R_ = 18.4 min) as a principal component and three minor anthraquinones (pseudopurpurin, rubiadin and nordamnacanthal) were identified. The purpurin elicited a stronger but not apoptosis-mediated antitumor effect in melanoma cells (A2058: 10^−6^–10^−5^ M: 90.6–64.1 %) than in normal fibroblasts (10^−6^–10^−5^ M: 97.6–84.8 %). The aqueous extract in equimolar concentrations showed the most potent cytotoxicity after 72 h incubation (A2058: 10^−6^–10^−5^ M: 87.4–55.0 %). All tested substances elicited chemorepellent effect in melanoma cells, while in MRC-5 fibroblasts, only the alizarin exhibited such a repellent character. Indices of chemokinesis measured by holographic microscopy (migration, migration directness, motility and motility speed) were significantly enhanced by alizarin and purpurin as well, while morphometric changes were weak in the two melanoma cell lines.

**Conclusions:**

Our results highlight the effective and selective inhibitory activity of purpurin towards melanoma cells and its possible use as a targeted anticancer agent. The anthraquinones of the cytotoxic extract are suggested to apply in drug delivery systems as an anticancer drug.

**Electronic supplementary material:**

The online version of this article (doi:10.1186/s12935-015-0271-4) contains supplementary material, which is available to authorized users.

## Background

Common madder (*Rubia tinctorum* L.) is a well-known, traditional medicinal plant. It contains substantial amount of anthraquinones in its root and rhizome. The plant has been used to dye textiles and as food colorant in many parts of the world since ancient times. Furthermore the crude extract of Rubia has been used in folk medicines as an anti-inflammatory, antibacterial and antifungal agent [1], or for the treatment of bladder and kidney stones, especially those consisting of calcium oxalate and calcium phosphate in the urinary tract [2–4].

**Fig. 1:**
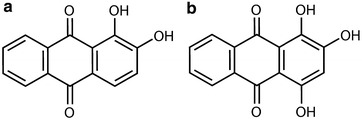
Chemical structure of the **a** alizarin and **b** purpurin

The pharmacologically important main components of *Rubia tinctorum* L. are di- and trihydroxyanthraquinones: alizarin (Fig. 1a), purpurin (Fig. 1b) and their derivatives, ruberythric acid (alizarin-primeveroside), pseudopurpurin and lucidin-primeveroside [3]. Furthermore, several other anthraquinones were identified as munjistin, pseudopurpurin, nordamnacanthal and lucidin [5, 6].

Several synthetic anthraquinones such as doxorubicin are one of the most effective agents for the treatment of different tumors in clinical practice; however, they have several drawbacks including lack of selectivity and consequently toxicity to normal cells [7]. Selective and significant antitumor actions could be achieved by direct inhibition of different tumor specific molecular targets (e.g. overexpressed receptors or biochemical processes). Application of drug delivery systems or drug targeting conjugates is another well-accepted strategy. In this case a delivery unit ensures the targeting and the internalization of a drug furnished conjugate via its receptors overexpressed on tumor cells [8–10].

In case of alizarin a selective cytostatic activity was reported towards primary bone cancers or tumors metastasized to bone by inhibiting the activation of ERK signaling and inducing cell cycle arrest in S phase [2]. Another study has reported the purpurin as an inhibitor of cell migration through blockade the leukotriene receptor (LTB2 receptor of leukotriene B4 (LTB_4_)) induced signaling [11], which pathway was shown to involve in e.g. prostate, ovarian, breast tumor progression [12–14].

For analytical characterization of the anthraquinone constituents of *Rubia tinctorum* L. and investigation of their effects on melanoma cell proliferation, adhesion and migration, transformed root cultures were used in our work. The application of genetically modified hairy root cultures provides several advantages. These cultures are genetically stable, have the ability to grow rapidly on media containing no growth regulators, and their secondary metabolite production reaches that of the corresponding plant or may exceed in some cases [15–17]. Previously we established a simple isocratic method to analyze the alizarin and purpurin contents of genetically transformed roots of *Rubia tinctorum* L. following an acidic hydrolysis [18]. One of the limitations of this acidic treatment is the possible formation of the mutagenic lucidin [19]. Derksen and his co-workers described methods of aqueous hydrolysis by native enzymes, which resulted in a suspension containing pseudopurpurin, munjistin, alizarin and nordamnacanthal without the formation of lucidin [5, 6].

Based on the above mentioned findings [2, 11] it is assumed that these anthraquinones or other components of Rubia extracts could be exploited for inhibition of tumor growth and progression as well as for design drug components of conjugates used in targeted tumor therapy.

In this present work the above described method of aqueous hydrolysis was applied to qualitatively characterize the anthraquinone contents of genetically transformed root cultures of *Rubia tinctorum* L. by HPLC and ESI–MS methods. The effects of the aqueous extract on the cell physiological responses (proliferation, cell adhesion/morphology and migration) in human cell lines representatives of metastatic tumors (melanomas) and a normal fibroblast model cell were investigated and compared to the activity of alizarin and purpurin. We aimed to evaluate tumor selective actions of Rubia-derived anthraquinones and to find an antiproliferative/cytotoxic anthraquinone as a drug component in a potential drug delivery system.

## Results

### Qualitative determination of the extract

**Fig. 2:**
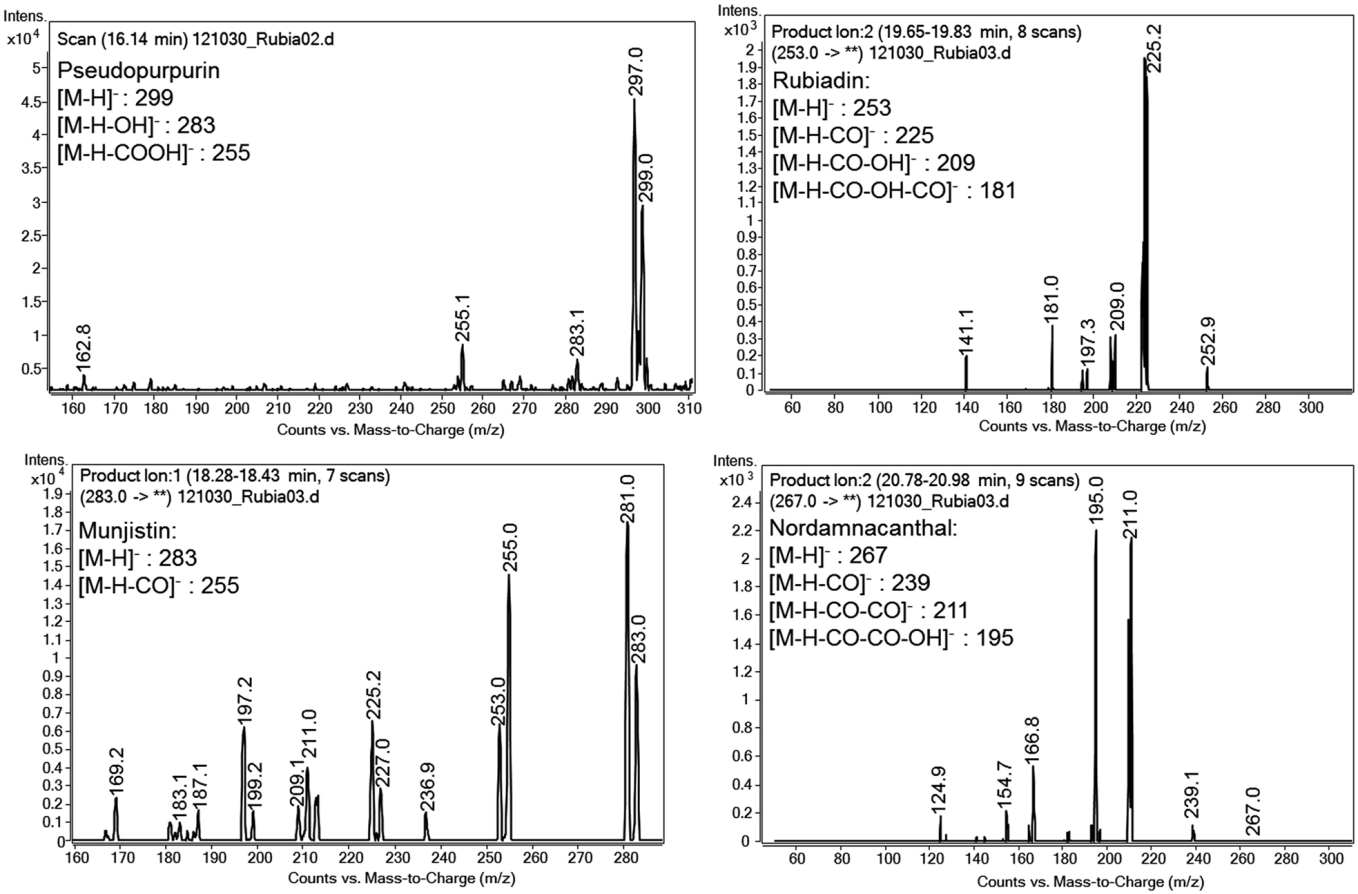
ESI-MS spectra of the anthraquinones identified in the aqueous extract of *Rubia tinctorum* L.

**Fig. 3:**
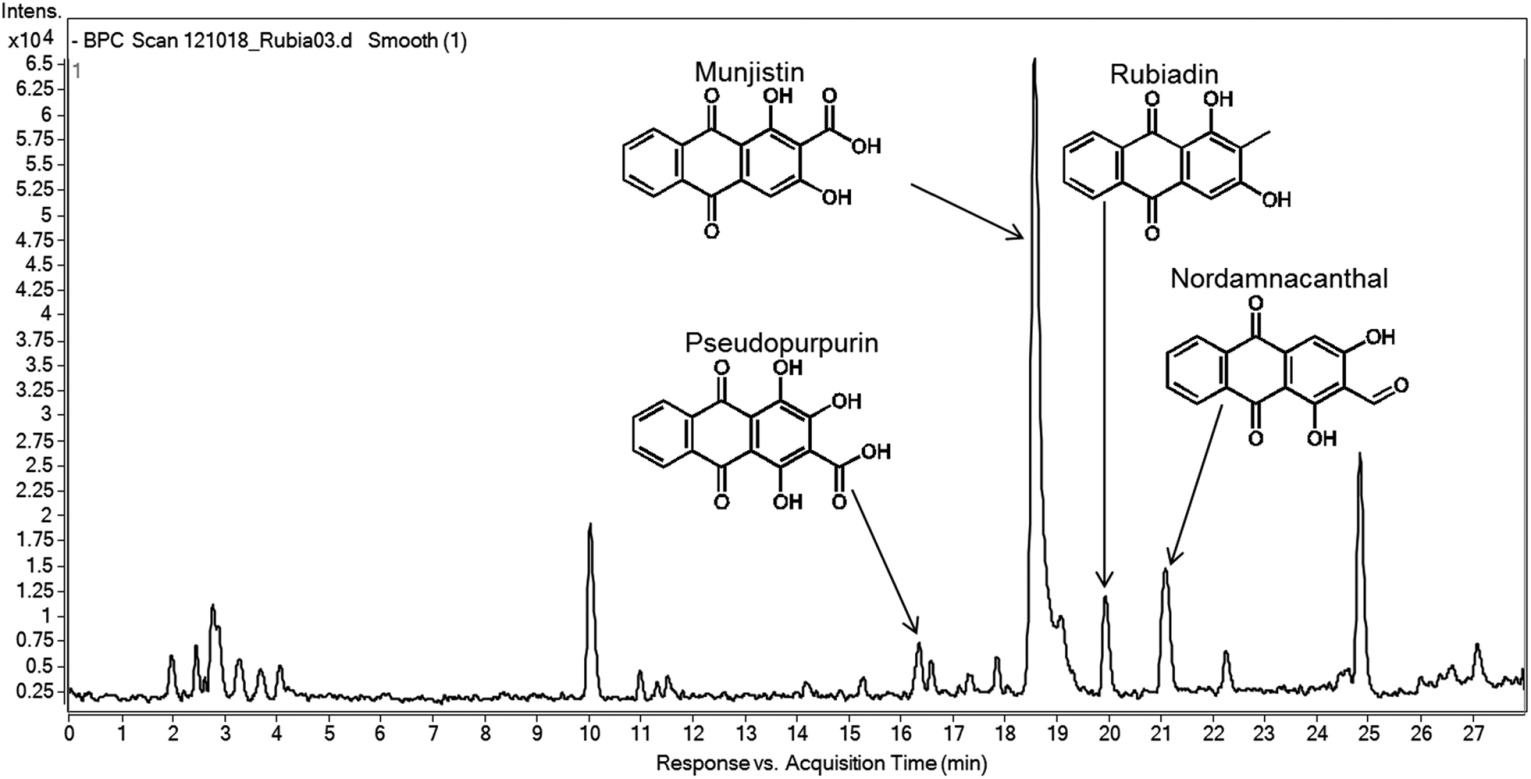
HPLC total ion chromatogram of the aqueous extract of *Rubia tinctorum* L.

The authentic standards alizarin and purpurin as well as the purified aqueous extract of *Rubia tinctorum* L. were analyzed by LC–MS/MS method (Fig. 2). Assuming that the anthraquinones of the extract ionize like the anthraquinone standards, the base peaks would correspond with the [M–H]¯ ions. For all of the studied anthraquinones, a loss of 28 amu was observed, corresponding to a loss of CO. In two cases a loss of 44 amu was detected, which agreed with the decarboxylation of the parent ion [M–H–CO_2_]¯. The data obtained were compared with data from literature [20, 21]. From this comparison, four peaks were tentatively identified as pseudopurpurin (*M*_r_ = 300, *t*_R_ = 16.1 min), munjistin (*M*_r_ = 284, *t*_R_ = 18.4 min), rubiadin (*M*_r_ = 254, *t*_R_ = 19.8 min) and nordamnacanthal (*M*_r_ = 268, *t*_R_ = 21.0 min) (Fig. 3).

### Antiproliferative/cytotoxic effects

**Fig. 4:**
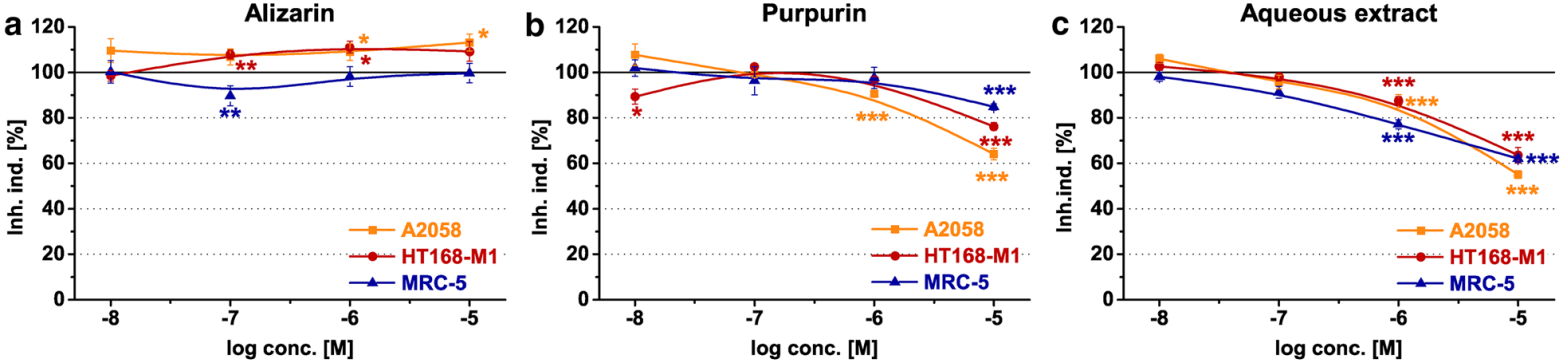
Long term growth inhibitory effects of **a** alizarin, **b** purpurin and **c** aqueous extract. Two different melanoma cell lines (A2058 and HT168-M1) and normal fibroblast cells (MRC-5) were applied as model cells and were incubated with the anthraquinones for 72 h. The ‘Inhibition index’ (Inh. ind.) is expressed as a percentage of the control. Data shown in the figure represent mathematical averages of six parallels and ± S.D. values. The level of significance is shown as follows: *p < 0.05; **p < 0.01; ***p < 0.001

To determine the long term antiproliferative/cytotoxic effect of alizarin, purpurin and the aqueous extract the cells were treated with the compounds for 48 and 72 h. The most significant effects could be observed after 72 h; therefore, only the results of the 72 h long treatments are presented below, and shown in Fig. 4, while the data of the 48 h long incubation are given in Additional file 1: Figure S1.

Seeding 10^4^ cells/well in a 96-well plate formed non-confluent layer after 24 h of culturing when the treatment occurred. In case of the cytotoxicity experiments, the cells were in the logarithmic phase of growth at the treatment.

The alizarin elicited a slight, but significant proliferation inducer effects in both melanoma cell lines with the same activity in 10^−7^–10^−5^ M range (A2058: 106.8–113.2 %, *p* < 0.05; HT168-M1: 107.9–109.3 %, *p* < 0.05) (Fig. 4a). In contrast, an inhibitory character of alizarin was shown in MRC-5 cells after 72 h in 10^−7^ M concentration (89.7 %, *p* < 0.01) (Fig. 4a).

The purpurin displayed a concentration and time dependent growth inhibitory effect in A2058 cells (10^−6^–10^−5^ M: 90.6–64.1 %, *p* < 0.001) (Fig. 4b). A weaker inhibitory effect of purpurin was detected in HT168-M1 cell line. Interestingly, this compound decreased the number of HT168-M1 cells both in the lowest (10^−8^ M: 89.3 %, *p* < 0.05) and highest concentration (10^−5^ M: 76.2 %, *p* < 0.001) tested in this experiment. In case of MRC-5 normal cells the purpurin displayed lower antiproliferative/cytotoxic effect than in melanoma cell lines and it was shown only in 10^−5^ M (84.8 %, *p* < 0.001) (Fig. 4b).

The treatment with the aqueous extract exhibited a strong decrease in the number of melanoma cells in 10^−6^–10^−5^ M concentration range, which activity was demonstrated already after 48 h (Additional file 1: Figure S1c). This inhibitory effect was more pronounced in A2058 (10^−6^–10^−5^ M: 87.4–55.0 %, *p* < 0.001) than in HT168-M1 cells (10^−6^–10^−5^ M: 87.5–63.7 %, *p* < 0.001) after 72 h (Fig. 4c). In MRC-5 cells a similar, concentration dependent antiproliferative/cytotoxic effect (10^−6^–10^−5^ M: 77.2–62.0 %, *p* < 0.001) was observed for this aqueous extract (Fig. 4c).

The effect of short term treatment (6 h) with the anthraquinones was also examined. The antiproliferative/cytotoxic effect of the compounds was detected by MTT-assay after washing out the substances from the cells and a further 72 h of culturing. In this experiment, the most effective concentrations (10^−6^, 10^−5^ M) of the long term cytotoxicity study were used. The results of the short term growth inhibitory effect are presented in Table 1.

**Table 1:** Short term growth inhibitory effects of alizarin, purpurin and aqueous extract

Compound	Conc. [M]	Inhibition index^a^ (%) (control = 100 %)		
		A2058	HT168-M1	MRC-5
Alizarin	10^−6^	98.3 ± 1.52	98.3 ± 1.46	98.4 ± 1.62
	10^−5^	103.8 ± 1.30	96.5 ± 1.61	104.6 ± 1.86
Purpurin	10^−6^	100.0 ± 1.45	100.8 ± 1.55	99.1 ± 1.12
	10^−5^	90.6*** ± 1.09	87.0*** ± 1.79	103.16 ± 2.14
Aqueous extract	10^−6^	93.3* ± 0.86	101.9 ± 1.24	102.7 ± 1.52
	10^−5^	84.5*** ± 1.06	116.1*** ± 1.39	100.3 ± 1.17

Two different melanoma cell lines (A2058 and HT168-M1) and normal fibroblast cells (MRC-5) were applied as model cells and were incubated with the anthraquinones for 6 h

The ‘Inhibition index’ is expressed as a percentage of the control. Data shown in the table represent mathematical averages of six parallels and ± S.D. values

The level of significance is shown as follows: * p < 0.05; *** p < 0.001

The alizarin proved to be neutral on the proliferation of all model cells. The purpurin significantly reduced the number of melanoma cells but only at 10^−5^ M concentration (A2058: 90.6 %, *p* < 0.001; HT168-M1: 87.0 %, *p* < 0.001); however, the purpurin showed a lower activity after 6 h treatment compared to its 72 h-long effect (A2058: Inh. Ind._6h_ = 90.6 % vs. Inh. Ind._72h_ = 64.1 %; HT168-M1: Inh. Ind._6h_ = 87.0 % vs. Inh. Ind._72h_ = 76.2 %). The purpurin had no influence on the proliferation of MRC-5 cells.

In case of the aqueous extract, a growth inhibitory effect was observed only in A2058 cells (10^−6^ M: 93.3 %, *p* < 0.05; 10^−5^ M: 84.5 %, *p* < 0.001), but this effect was less pronounced than its long-term activity (10^−6^–10^−5^ M: Ind._6h_ = 93.3–84.5 % vs. Inh. Ind._72h_ = 87.4–55.0 %). The extract had a slight, but significant proliferation inducer activity for HT168-M1cells (10^−5^ M: 116.1 %, *p* < 0.001) and a neutral effect for MRC-5.

### Apoptotic effects

The apoptotic effect of alizarin, purpurin and the aqueous extract was analyzed by flow cytometry using annexin V-FITC labeling. The compounds were applied in 10^−5^ M for 72 h, since in most of the cases, this concentration and incubation time displayed the highest antiproliferative/cytotoxic effect.

**Fig. 5:**
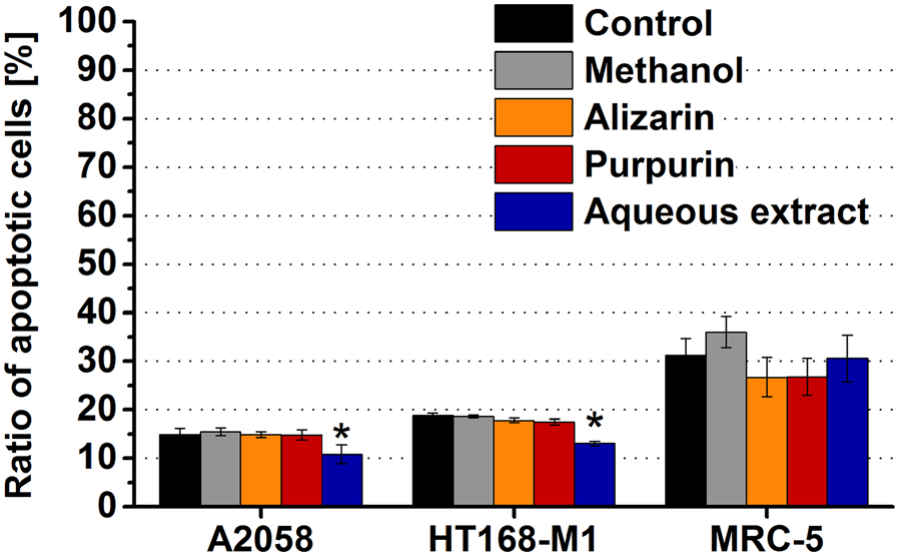
Apoptotic effects of alizarin, purpurin and aqueous extract. Two different melanoma cell lines (A2058 and HT168-M1) and normal fibroblast cells (MRC-5) were applied as model cells and were incubated with the anthraquinones (10^−5^ M) for 72 h. The ‘Ratio of apoptotic cells’ is expressed as a percentage of viable cells measured by flow cytometry. Data shown in the figure represent mathematical averages of two parallels and ± S.D. values. The level of significance is shown as follows: *p < 0.05

The alizarin and the purpurin failed to exert apoptotic effect in all model cells (Fig. 5). Nearly the same ratio of apoptotic melanoma cells was detected in the alizarin (A2058: 14.9 %; HT168-M1: 17.8 %) or purpurin (A2058: 14.8 %; HT168-M1: 17.5 %) treated groups than in case of the methanol-treated control population (A2058: 15.4 %; HT168-M1: 18.6 %). While, both compounds slightly decreased the percentage of the apoptotic MRC-5 cells (alizarin: 26.7 %, purpurin: 26.8 %) comparing to the identical control (36.0 %) (Fig. 5). The aqueous extract exhibited an opposite profile; it proved to be less apoptotic for the melanoma cells (A2058: 10.8 %, *p* < 0.05; HT168-M1: 13.1 %, *p* < 0.05) comparing to the control (A2058: 13.7 %; HT168-M1: 18.3 %) and had no effect on the number of the annexin V positive MRC-5 cells (30.6 vs. 31.2 %) (Fig. 5).

### Cell adhesion modulator properties

The cell adhesion curves of our model cells display different cell type specific shapes, but in all cases three main phases of the adhesion kinetic can be differentiated. The initial phase (first 3 h after the cell inoculation) is the rapid adhesion characterized by a steep increase in the Cell index (CI) values. In the following phase (Δt ~ 3–20 h) the spreading of the cells accompanied by a slower, gradual increasing tendency of CI values. In long term (after 20–25 h) due to the cell proliferation a greater CI change can be observed (Additional file 1: Figure S2).

The adhesion of the cells and the effects of the anthraquinones on it were described by the slope of Delta CI calculated for a 3 h’ time interval (Table 2).

**Table 2:** Adhesion modulator effect of the alizarin, purpurin and the aqueous extract

Compound	Conc. [M]	Slope^a^ (%) (control = 100 %)		
		A2058	HT168-M1	MRC-5
Alizarin	10^−8^	88.9 ± 6.74	100.4 ± 3.05	99.3 ± 4.96
	10^−7^	100.6 ± 4.01	104.3 ± 3.16	104.7 ± 0.82
	10^−6^	110.8 ± 6.38	110.4 ± 1.37	100.2 ± 2.44
	10^−5^	90.4 ± 8.7	107.3 ± 6.51	95.5 ± 7.08
Purpurin	10^−8^	85.6 ± 4.36	93.6 ± 4.91	102.1 ± 5.44
	10^−7^	97.6 ± 1.86	101.0 ± 3.42	103.7 ± 2.22
	10^−6^	95.2 ± 14.01	97.8 ± 15.16	109.6 ± 2.4
	10^−5^	115.6 ± 6.56	110.2 ± 4.03	93.8 ± 5.14
Aqueous extract	10^−8^	99.3 ± 4.96	102.1 ± 5.44	105.4 ± 1.64
	10^−7^	104.7 ± 0.82	103.7 ± 2.22	104.5 ± 1.99
	10^−6^	100.2 ± 2.44	109.6 ± 2.4	98.4 ± 4.25
	10^−5^	95.5 ± 7.08	93.8 ± 5.14	91.7* ± 2.58

Two different melanoma cell lines (HT168-M1) and normal fibroblast cells (MRC-5) were applied as model cells

Data shown in the table represent mathematical averages of three parallels and ± S.D. values

The level of significance is shown as follows: * p < 0.05

^a^The slope values are expressed in percentage of the control and describe the changing rate of Delta CI in first 3 h’ time interval of cell adhesion

In a separate experiment with xCELLigence System, the growth rate of the melanoma cells were monitored for at least 72 h after inoculating 10^4^ cells/well. The growth curves showed that both melanoma cell lines were in log phase. The detected CI parameter was continuously increased as the melanoma cell number was increased over the time (Additional file 1: Figure S3). Based on the growth curves of A2058 and HT168-M1 cells, 33 and 27 h doubling times were calculated by RTCA 1.2 software of the xCELLigence SP (Additional file 1: Figure S3).

Neither the pure anthraquinones nor the aqueous extract had any significant influence on the cell adhesion of melanoma cells; however a clear positive tendency can be observed in case of pure compounds. The alizarin in 10^−6^ M (A2058: 110.8 %; HT168-M1: 110.4 %), while the purpurin in 10^−5^ M (A2058: 115.6 %; HT168-M1: 110.2 %) slightly induced the adhesion of both melanoma cells with nearly the same activity. In case of MRC-5 fibroblasts only the purpurin had a weak, not significant, positive effect on cell attachment in 10^−6^ M (109.6 %) (Table 2). The aqueous extract elicited a slight, but significant decreasing effect (10^−5^ M: 91.7 %, *p* < 0.05) on MRC-5 adhesion (Table 2).

### Chemotactic effects

**Fig. 6:**
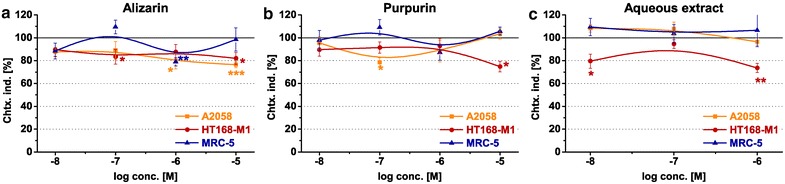
Chemotactic characters of the **a** alizarin, **b** purpurin and the **c** aqueous extract. Two different melanoma cell lines (A2058 and HT168-M1) and normal fibroblast cells (MRC-5) were applied as model cells. The ‘Chemotaxis index’ (Chtx. ind.) is expressed as a percentage of the control. Data shown in the figure represent mathematical averages of eight parallels and ± S.D. values. The level of significance is shown as follows: *p < 0.05; **p < 0.01; ***p < 0.001

The comparison of chemotactic response of our model cells induced by the pure anthraquinones and the aqueous extract is shown on Fig. 6.

Both of the alizarin and purpurin elicited chemorepellent responses in both melanoma cell lines; however, in different concentration range. The chemorepellence of alizarin was observed in 10^−6^–10^−5^ M concentrations (79.3–76.4 %, *p* < 0.05 and *p* < 0.001, respectively) in case of A2058 cells, and in 10^−7^–10^−5^ M for HT168-M1 cells (89.6–81.9 %, *p* < 0.05) (Fig. 6a). The purpurin induced negative chemotactic responses of HT168-M1 in 10^−7^ M (78.6 %) in A2058 cells, and in 10^−5^ M (74.8 %) (Fig. 6b). In MRC-5 cells the alizarin also proved to be significantly repellent in 10^−7^ M (78.9 %, *p* < 0.01) (Fig. 6a), while the purpurin was neutral in the entire concentration range (Fig. 6b).

The aqueous extract had chemorepellent effect only in HT168-M1 cells both at 10^−6^ M and 10^−8^ M concentrations (79.5 %, *p* < 0.05 and 73.6 %, *p* < 0.01, respectively), while in case of A2058 and MRC-5 it elicited no chemotactic effect (Fig. 6c).

### Chemokinetic effects

Cell-tracking experiments accomplished by holographic microscopy represent a dedicated way to analyze chemokinetic activities in complexity of cellular migratory responsiveness. The analyzed four parameters (‘Avg. Migration’, ‘Avg. Motility’, ‘Avg. Migration directness’ and ‘Avg. Motility speed’) present information about the most important, chief characteristics (distance, orientation and speed) of cellular locomotion. As the time-frame of experiments was rather wide (2 h) our goal was to evaluate not only the above mentioned parameters but also test whether the slopes of the corresponding graphs show any changes by the time.

Our results show that the both alizarin and purpurin (both substances were tested in the optimal 10^−5^ M concentration) had significantly positive, cell locomotor inducer effects in both melanoma cell lines (Tables 3, 4). Only ‘Avg. Migration directness’ (migration/motility) failed to fit to the trend in the case of alizarin in A2058 cells. Values of slopes had no such a general and concordant relation to the migratory responses. This means that while good correlations were observed between the alizarin and purpurin induced migratory responses (e.g. purpurin—‘Avg. Migration’, ‘Avg. Migration directness’ ‘Avg. Motility’; alizarin—‘Avg. Motility’, ‘Avg. Motility speed’) and their positive slope trends in A2058 cells (Table 3), no such clear positive slope trend was observed in HT168-M1 cells (Table 4).

**Table 3:** Migratory behavior of A2058 melanoma cells detected by holographic microscopy

A2058	Methanol	Alizarin (10^−5^ M)	Purpurin (10^−5^ M)
Avg. Migration (μm)			
Mean	5.347 ± 0.16	8.719 ± 0.25**	22.667 ± 0.82***
Slope	0.034 ± 6.82E−4	0.056 ± 7.03E−4	0.170 ± 0.01***
R	0.955	0.981	0.933
Adj. R-square	0.912	0.963	0.870
Avg. migration directness (migration vs motility)			
Mean	0.110 ± 0.01	0.100 ± 0.01	0.196 ± 0.01**
Slope	−6.40E−4 ± 7.83E−5	−7.14E−4 ± 8.38E−5	−2.30E−4 ± 6.79E−5**
R	0.468	0.482	0.214
Adj. R-square	0.215	0.229	0.042
Avg. motility (µm)			
Mean	75.229 ± 2.63	125.627 ± 4.94**	104.172 ± 3.99*
Slope	0.587 ± 0.01	1.099 ± 0.01***	0.890 ± 0.01**
R	0.998	0.997	0.998
Adj. R-square	0.996	0.995	0.996
Avg. motility speed (µm/h)			
Mean	79.931 ± 1.14	134.900 ± 2.44**	104.630 ± 4.75*
Slope	0.063 ± 0.01	0.279 ± 0.03***	0.045 ± 0.06
R	0.248	0.514	0.042
Adj. R-Square	0.057	0.261	0.002

Effect of treatment with alizarin (10^−5^ M) and purpurin (10^−5^ M)

Data shown in the table represent variables and ± S.D. values calculated for 50 cell/group in 240 consecutive frames

The ‘Mean’ values are expressed as dimensions (μm, μm/h) shown in the sub-headers of the table

The slope values are expressed as dimensionless values to describe the changing rate of Mean values in the 2 h’ time interval of treatments

R and Adj. R-Square represent data of linear regression analysis to describe trends of variables belonging to the parameters calculated

The level of significance is shown as follows: * p < 0.05; ** p < 0.01; *** p < 0.001

**Table 4:** Migratory behavior of HT168-M1 melanoma cells detected by holographic microscopy

HT168-M1	Methanol	Alizarin (10^−5^ M)	Purpurin (10^−5^ M)
Avg. Migration (μm)			
Mean	8.870 ± 3.65	26.651 ± 10.65***	15.490 ± 6.14***
Slope	0.052 ± 4.54E−4	0.140 ± 0.01	0.086 ± 0.01
R	0.991	0.920	0.976
Adj. R-square	0.982	0.846	0.953
Avg. migration directness (migration vs. motility)			
Mean	0.127 ± 0.01	0.189 ± 0.01**	0.146 ± 0.01
Slope	6.55E−4 ± 8.10E−5	8.23E−4 ± 7.26E−5	7.52E−4 ± 7.89E−5
R	0.464	0.524	0.464
Adj. R-square	0.212	0.346	0.272
Avg. motility (µm)			
Mean	96.346 ± 3.49	184.515 ± 7.38***	118.951 ± 4.21*
Slope	0.778 ± 0.01	1.643 ± 0.01	0.937 ± 0.01
R	0.998	0.999	0.997
Adj. R-square	0.997	0.998	0.997
Avg. motility speed (µm/h)			
Mean	96.609 ± 1.46	188.513 ± 3.94***	115.551 ± 2.05
Slope	0.056 ± 0.02	0.199 ± 0.05	0.033 ± 0.03
R	0.172	0.227	0.074
Adj. R-square	0.025	0.047	0.001

Effect of treatment with alizarin (10^−5^ M) and purpurin (10^−5^ M)

Data shown in the table represent variables and ± S.D. values calculated for 50 cell/group in 240 consecutive frames

The ‘Mean’ values are expressed as dimensions (μm, μm/h) shown in the sub-headers of the table

The slope values are expressed as dimensionless values to describe the changing rate of Mean values in the 2 h’ time interval of treatments

R and Adj. R-Square represent data of linear regression analysis to describe trends of variables belonging to the parameters calculated

The level of significance is shown as follows: * p < 0.05; ** p < 0.01; *** p < 0.001

### Morphometry

Holographic microscopy detects morphological characteristics of cells with high sensitivity and contributes to distinguish cytotoxic and apoptotic processes. The available parameters calculated are grouped as basic (‘Avg. Area’, ‘Avg. Thickness’, ‘Avg. Volume’) and complex (derived) ones (see the list and definitions in Sect. “Methods”).

Results showed that no basic parameter was influenced by alizarin or purpurin as a cytotoxic or apoptotic substance in HT168-M1 cells. Moreover, in the case of A2058 cells alizarin could induce positive effects even in two variables (‘Avg. Area’ and ‘Avg. Volume’). Nevertheless, in the case of complex parameters A2058 cells proved to be more sensitive as significant decreases were detected in slopes of variables ‘Avg. Eccentricity’ and ‘Avg. Hull convexity’ (the later parameter was also significantly decreased in purpurin treated group, too) (Additional file 2: Tables S1–S4).

## Discussion

In the present study the alizarin, purpurin and the aqueous extract were investigated in terms of selective activity on proliferation, cell adhesion and migration of tumor—melanoma—cells. On the other hand the composition of the aqueous extract was analyzed and the molecular structure of the anthraquinones was evaluated with the aim to find an antitumor compound, which has a suitable functional group for using it as an effective compound in conjugate(s) of drug targeting.

### Determination of constituents of the aqueous extract

In our study the method of hydrolysis by endogenous enzymes developed by Derksen et al. was used to prepare an extraction of *Rubia tinctorum* L. hairy root culture. They could detect pseudopurpurin, munjistin, alizarin and nordamnacanthal in the plant suspension [6]. In this study, the qualitative analysis of the extract was performed by LC–MS/MS method. The munjistin (1,3-dihydroxyanthraquinone-2-carboxylic acid) was identified as the main component. In addition, the detailed analysis of the extract led to the identification of three minor anthraquinones: pseudopurpurin (1,2,4-trihydroxyanthraquinone-3-carboxyl acid), rubiadin (1,3-dihydroxy-2-methylanthraquinone) and nordamnacanthal (1,3-dihydroxyanthraquinone-2-al).

The main water soluble anthraquinones in madder roots are the glycosides (ruberythric acid and lucidin primeveroside) and the anthraquinone carboxylic acids (munjistin and pseudopurpurin). Similarly to the above mentioned study, our aqueous extract—as it was expected—did not contain the mutagenic lucidin primeveroside or lucidin. The glycosides can be easily hydrolyzed by endogenous enzymes to their aglycons (alizarin and lucidin), after which, for example lucidin can be further converted to non-genotoxic nordamnacanthal. The alizarin and nordamnacanthal are insoluble in water, which could explain why the alizarin was not found and only in small amount of nordamnacanthal was detected in our aqueous extract. In contrary, Derksen and co-workers could identify the alizarin and nordamnacanthal as the main components of the extract, but their extraction method was different as that was used in the present study. The initial extraction with distilled water was followed by addition of tetryhydrofuran-water-formic acid solution to dissolve the precipitated anthraquinone agclycons. The different durations of extraction (45 or 90 min vs. 24 h) might also result in suspensions containing different anthraquinone derivatives or diverse ratios of these molecules [5, 6]. For example, the rubiadin in our extract might be also converted from lucidin by endogenous enzymes as a result of longer stirring [22].

### Tumor growth inhibitory effects of the alizarin, purpurin and aqueous extract

In our experiments on metastatic melanoma cell lines the alizarin and purpurin were included as representatives of Rubia-derived dihydroxy- and trihydroxyanthraquinones. In contrast to the previous results obtained with colon- [23], breast carcinoma and osteosarcoma cell lines [2], in our study the alizarin did not have any antiproliferative or cytotoxic effect in melanoma cells. It was suggested that depending on the tumor type (osteosarcoma vs. prostate carcinoma cell line) the alizarin might exert antitumor actions with different effectiveness [2]. The antiproliferative activity of the anthraquinones has been also shown to be determined by the number of OH groups. The compounds with more OH group were reported to be more effective [23]. Our results on melanoma cells—the neutral effect of dihydroxyanthraquinone alizarin and the tumor growth inhibitory activity of the purpurin (trihydroxyanthraquinone)—are in good agreement with the findings cited above.

In our present study the aqueous extract proved to be the most effective antiproliferative/cytotoxic agent in melanoma cells. Limited data are available in the literature about the antiproliferative or cytotoxic effects of the anthraquinones which were identified in this extract. Few studies have demonstrated that the nordamnacanthal or its analogue (damnacanthal) inhibited the proliferation of various cell lines [24, 25]. Although, there is no information about the direct tumor growth inhibitory action of rubiadin but its significant phototoxicity has been observed in a breast cancer cell line [26]. The more potent antitumor activity of the extract in equimolar concentration than the pure anthraquinone compounds can be explained (1) by the possible additive actions of recognized anthraquinones, or (2) by other unknown minor compounds of the extract.

The MTT assay, which was used to determine the tumor inhibitory effect of the anthraquinone compounds, could provide information about the number of the viable cells by detecting the NADH-dependent dehydrogenase activity, but not about the form of cell death. In order to explore the mechanism of their antitumor effects, an apoptosis measurement was carried out. It was shown that anthraquinones failed to induce apoptosis, so their antitumor effects were mediated by unknown mechanisms (e.g. altering the cell cycle [2]) rather than inducing apoptosis.

In most of the cases it took at least 48 h for the anthraquinones to exert their significant antitumor activity on melanoma cells and this activity became even more pronounced for 72 h. This lag in their effect could be explained by the cell cycle length (A2058: 33 h; HT168-M1: 27 h) and the non-synchronous division of the melanoma cells. Although, the A2058 cell line exhibits lower proliferation rate and metastatic capacity [27] than the HT168-M1 cell line, the A2058 cells appeared to be more sensitive toward the long-term antiproliferative/cytotoxic effects of purpurin and aqueous extract than the HT168-M1 cells. So other factors, like the confluence of the cell culture, or cellular uptake of the anthraquinones were also taken into consideration. It was reported that efficacy of a chemotherapeutic agent on tumor growth highly depends on the confluence or the phases of cell growth. For example, anthracyclines (e.g. doxorubicin) had lower cytotoxic activity in cells of stationary growth phase compared with exponentially growing cells [28]. As it was demonstrated in a real time measurement, the cells were in a logarithmic growth phase during 72 h. The anthraquinone-resistance induced by the noncycling state (stationary phase) of cells could be excluded. Nevertheless, the antitumor efficacy of the anthraquinones in stationary, no- or slowly growing cells would be interesting to investigate. The efficacy of the chemotherapeutic drugs in noncycling cells basically depends on the mechanism of their effect, which is still an open question in case of the Rubia-derived anthraquinones.

Regarding the internalization of the anthraquinones it was found that, less than 10 % of melanoma cells could internalize the alizarin after 72 h long incubation time. However, the cellular uptake of purpurin was higher, but only about 60 % of melanoma cells were “anthraquinone positive” (Additional file 1: Figure S4). The difference in the efficacy of the compounds could be also determined by their internalization. The increased cellular uptake seemed to enhance the anti-tumor activity. The order of the internalization rate and the antitumor activity of the compounds was the same: alizarin ≪ purpurin < aqueous extract.

However, the tumor growth inhibitory effect of long-term treatment manifested after 48 h, the washing out of the anthraquinones after 6 h-long exposure could still cause a slighter but significant antiproliferative effect. This means that the effect of the compounds appeared to be irreversible for melanoma cells.

### Activities of alizarin, purpurin and aqueous extract on the cell adhesion and migration

The metastatic spreading of tumor cells from the primary site is a crucial aspect of tumor progression and can significantly affect the effectiveness of the targeted tumor therapy. The altered cell adhesion and migration of tumor cells are critical for the tumor cells to invade the surrounding tissues, to migrate into a distant site and to attach and form a new colony [29].

Although the Rubia-derived anthraquinones could not have significant cell adhesion modulator effect, they could interfere with the migration of metastatic melanoma cells. Both of the pure anthraquinones induced the repellent responses of both A2058 and HT168-M1. Our present results are in harmony with a study on the indirect blocking effect of purpurin in LTB_4_ induced chemotactic reaction of CHO cells [11].

Characterization of anticancer substances requires a detailed analysis of cell physiological responses in their complexity. Locomotor activity and alteration of morphological parameters are considered as chief tumor specific features to be investigated as prognostic markers of the candidate drugs. In our present work both alizarin and purpurin proved to possess characteristic chemorepellent effects as well as chemokinesis enhancer characters as both length of tracks (‘Avg. Migration’ and ‘Avg. Motility’) and speed of migrating cells were increased by the treatments. The complex analysis of migratory behavior shows that application of the two compounds is more advantageous as they potentially facilitate cell accumulation in primary tumors and not spreading as metastatic ones. In respect of morphometry—despite the fact that a number of parameters were examined—our results are more moderate. Nevertheless, the data obtained support our conclusion detailed before that the two anthraquinone derivatives have no significant apoptotic effects in the backgrounds of their cytotoxicity.

### Evaluation of the suitability of anthraquinones for targeted tumor therapy

Investigation of the toxic effect of anthraquinones on the normal cells is also an important issue regarding the design of the targeted therapy. This consideration justifies that MRC-5 normal human fibroblast cells were also involved in our study. Notably, the normal fibroblasts proved to be more resistant to purpurin in all in vitro assays, which suggests that purpurin has a selective toxicity and chemorepellence on melanoma cells. There was a good correlation between the uptake and the efficacy of purpurin obtained in melanoma and MRC-5 cells. In case of MRC-5 cells, the lower antiproliferative/cytotoxic activity of purpurin could be due to its low internalization rate, while the increased cellular uptake was associated with more pronounced antitumor activity in melanoma cells. Comparing the doubling times of the melanoma cells (A2058: 33 h; HT168-M1: 27 h) and MRC-5 cells (in publications rather different values were reported: 27 h [30], 34 h [31], 40 h [32]) there was not a significant difference, which could have explained the resistance of MRC-5 to purpurin. Nevertheless, such a remarkable difference in the cellular uptake of the purpurin by melanoma cells and MRC-5 cell line might suggest that the purpurin acts on a cell surface receptor expressed differently in tumor vs. normal cells.

This tumor selective activity of purpurin is underlined by the fact that other anthraquinones including emodin (trihydroxyanthraquinone) and mollugin (anthraquinone related compound) were reported to evoke a less or no cytotoxic effect in cells derived from normal breast tissue [33, 34].

In contrast to purpurin the growth inhibitory activity of aqueous extract was not selective to tumor cells; it reduced the cell number as well as the cell adhesion of normal fibroblasts. Some studies have reported that the rubiadin exerted strong genotoxicity as well as played a critical role as an initiator of carcinogenesis [35, 36]. It is possible that the non-selective damaging effect of aqueous extract is due to one of its genotoxic anthraquinone components. It is important to emphasize that the alizarin and purpurin are free of this type of activity [19, 37], while no data have been found yet about the genotoxicity of munjistin, pseudopurpurin or nordamnacanthal in the literature.

Our results suggest that the purpurin can exhibit selective anticancer activity and by inducing chemorepellent migratory response of melanoma cells it can interfere with tumor progression. These results indicate that the purpurin might be developed as a targeted anticancer agent acting on melanoma cell specific molecular target.

Although, the aqueous extract failed to exert tumor selective activity but the identification of munjistin, pseudopurpurin and nordamnacanthal might be promising agents for targeted therapy. Since these anthraquinone derivatives possess eligible moieties (carboxyl or aldehyde groups) they could be suitable candidates to be applied in drug delivery conjugates as an anticancer drug. Nevertheless, even more studies are required to verify the potential anticancer activity of the three individual anthraquinones composing aqueous extracts reported now as well as to exclude their genotoxicity.

## Conclusions

In summary, we showed that the purpurin, a trihydroxyanthraquinone and an aqueous extract from transformed root culture of *Rubia tinctorum* L. had tumor growth inhibitory activity in melanoma cell lines with different metastatic ability, and their effects proved to be irreversible and unrelated to apoptosis induction. The tested anthraquinones showed chemorepellent character and chemokinesis inducer effect in A2058 and HT168-M1 melanoma cells. Furthermore, the purpurin elicited significantly lower activity or neutral effect on proliferation and chemotaxis of normal fibroblast cells compared to the tumor model cells. These tumor selective properties of purpurin enable it to be used as targeted anticancer agent acting on a tumor cell specific molecular mechanism. The detailed analysis of aqueous extract led to the identification of munjistin as the principal component and three minor anthraquinones (pseudopurpurin, rubiadin, and nordamnacanthal). Based on the cytotoxic effect of the aqueous extract and the identification of anthraquinone derivatives possessing suitable moieties for coupling them to a peptide type targeting unit, our present results suggest the application of these anthraquinones in the tumor drug delivery conjugates as anticancer drugs.

## Methods

### Materials

#### Plant material: hairy root culture

The seeds of *Rubia tinctorum* L. were originated from VILAR Botanical Garden, Moscow. Transformed root cultures of *Rubia tinctorum* L. were obtained by their inoculation with *Agrobacterium rhizogenes* (strain R-1601). The hairy roots were cultured in liquid Gamborg B5 medium [38, 39] in Erlenmeyer flasks, in a Certomat BS-4 programmable incubation shaking cabinet (Braun Biotech International, Melsungen, Germany) at 100 rpm, under 23 ± 2 °C, in the dark.

#### Test substances

The tested substances were (1) pure anthraquinone compounds: alizarin (1,2-dihydroxy-9,10-anthraquinone) (Fig. 1a) and purpurin (1,2,4-trihydroxy-9,10-anthraquinone) (Fig. 1b) (Irvine, CA, USA) and (2) an aqueous extract of sample of *Rubia tinctorum* L. hairy root culture.

The purpurin and alizarin were solved in methanol to prepare the stock solution (2 × 10^−3^ M). Serial dilution ranged from 10^−8^ to 10^−5^ M were prepared from the stock in complete cell culture medium of each model cells. Each dilution has a control, where the identical ratio of methanol solved in complete cell culture medium.

The total concentration of anthraquinones of aqueous extract was expressed as a relative concentration correlated to the concentration of standard purpurin. The aqueous extract was further diluted to equimolar concentration (10^−8^–10^−5^ M) of pure anthraquinones in complete cell culture medium.

### Phytochemical analysis

#### Preparation of extract

Lyophilized (Christ Alpha 1-4 lyophilizator, Braun, Melsungen, Germany), and powdered material (100 mg) obtained from *Rubia tinctorum* L. hairy root culture was extracted with 50 ml of ultrapure water (purified with Millipore (Billerica, MA, USA) Direct-Q5 equipment) by stirring (100 rpm) overnight at room temperature.

#### Purification of extract

After filtration, 5 ml of the extract was passed drop-wise through solid phase extraction (SPE) cartridges (Supelclean LC-8, 3 ml; Supelco, Bellefonte, PA, USA) previously activated with methanol (5 ml) then water (5 ml). The fraction containing the anthraquinones was eluted from the cartridges with 2.5 ml of methanol.

#### Qualitative determination by LC–MS/MS

Compounds were separated on a LUNA octyl (5 μm) reversed phase column (250 × 4.6 mm I.D.; Phenomenex, Torrance, CA, USA). The column temperature was 25 °C and the injection volume 10 µl. The mobile phase was a linear gradient of acetonitrile—20 mM ammonium formate (pH 3.0) 15 → 100 (v/v). The flow-rate was 1 mL/min. Analysis was performed in negative ion mode on an Agilent 6410 Triple Quad LC/MS system using electrospray ionization. The injection volume was 10 μL. By solvent splitting, 40 % eluent was allowed to flow into the mass spectrometer. The conditions of the LC–MS/MS analysis were as follows: drying gas (N_2_) temperature 350 °C, drying gas flow rate 9 l/min, nebulizer pressure 45.0 psi (N_2_), fragmentor voltage 120 V, capillary voltage 4000 V, scan range from *m/z* 50 to 700 at collision energy of 15 or 20 eV depending on the molecular structure.

### Analysis of cell biological activities

#### Model cells

The effects of the Rubia-derived anthraquinones were evaluated in A2058 and HT168-M1 human melanoma cell lines with different metastatic potency. A2058 cells derived from a brain metastases shows high metastatic capacity [40]. HT168-M1 line is an in vivo selected, more metastatic derivative of A2058 [41]. The MRC-5 normal fibroblast cells derived from normal lung tissue were used as control [42].

Cultures of A2058 and HT168-M1 were maintained in RPMI 1640 (Sigma Ltd. St. Louis, MO, USA) containing 10 % FCS (Lonza Group Ltd., Switzerland), l-glutamine (2 mM), 100 µg/ml penicillin/streptomycin (Gibco^®^/Invitrogen Corporation, New York, NY, USA) and the MRC-5 cells with generation number between 18 and 20 were cultured in DMEM (Sigma Ltd. St. Louis, MO, USA) containing 10 % FCS (Lonza Group Ltd., Switzerland), l-glutamine (2 mM), 100 µg/ml penicillin/streptomycin (Gibco^®^/Invitrogen Corporation, New York, NY, USA) and 1 % non-essential amino acids (Gibco^®^/Invitrogen Corporation, New York, NY, USA) at 37 °C in a humidified 5 % CO_2_ atmosphere.

#### Cytotoxicity analysis

The long term antiproliferative/cytotoxic effects of anthraquinones and aqueous extract were determined by 3-(4,5-dimethylthiazol-2-yl)-2,5-diphenyltetrazolium bromide assay (MTT assay).

After seeding the cells (10^4^ cells/well) on 96-well plates and culturing them for 24 h, the cells were treated with substances using 10^−8^–10^−5^ M concentration range. After 48 h and 72 h incubation time the MTT (3-(4,5 dimethylthiazol-2-yl)-2,5-diphenyltetrazolium; Sigma Ltd., St. Louis, MO, USA) solution was added to each sample at 0.5 mg/ml final concentration.

After 4 h incubation with the MTT reagent the culture medium was removed and the reaction products, formazan crystals were dissolved in 100 μL dimethyl sulfoxide (DMSO). The optical density (OD) of the formazan solution was measured at 540 nm (OD_540_) and 620 nm (OD_620_) by ELISA reader (Labsystems Multiskan MS, Helsinki, Finland).

In order to evaluate whether the compounds have reversible antiproliferative/cytotoxic character the anthraquinones were removed from the cells after 6 h of incubation. The cells were washed twice with serum-free medium and cultured for a further 72 h in the serum-containing medium [43]. After this cell culturing period the MTT assay was carried out as described above.

Identical points of the concentration course study represent average of 6 parallel measurements. The decrease in the number of cells due to the anthraquinones (A) was normalized to the identical control (Ctrl) and this value was given as ‘Inhibition index’ (Inh. ind.) in percent.

1 $$ {\text{Inh}}.{\text{ ind }}\left[ \% \right] = \left( {{{(\overline{OD}_{540,A} - \overline{OD}_{620,A} )} \mathord{\left/{\vphantom {{(\overline{OD}_{540,A} - \overline{OD}_{620,A} )} {(\overline{OD}_{540,Ctrl} - \overline{OD}_{620,Ctrl} )}}} \right. \kern-0pt} {(\overline{OD}_{540,Ctrl} - \overline{OD}_{620,Ctrl} )}}} \right) \times 100 $$

#### Apoptosis assay

The apoptotic effect of the anthraquinones and the aqueous extract on our model cells was measured by FACSCalibur flow cytometer (Becton–Dickinson, San Jose, CA, USA) after staining with annexin V-FITC (Becton–Dickinson, San Jose, CA, USA).

Prior the treatment with the compounds, the cells were seeded on 12-well plates in 10^5^ cells/well density and cultured for 24 h. The compounds were added to the cells in 10^−5^ M concentration and the incubation time was 72 h (as in case of the long term cytotoxicity analysis). For the annexin V-staining the supernatant were removed and TrypLE reagent composed of recombinant cell-dissociation promoting enzymes (Thermo Fisher Scientific, Waltham, MA, USA) was added to the cells. The effect of the TypLE was stopped after 3 min by adding fresh serum-containing cell culture medium. The cells were transferred to FACS-tubes and after a centrifugation and a washing step with PBS (phosphate-buffered saline pH = 7.2) the annexin binding buffer (Becton–Dickinson, San Jose, CA, USA) was added to resuspend the cells. Before labeling with annexin V-FITC, the cells were measured by a flow cytometer for controlling the autofluorescence of the cells in case of each treatment group. Then, the cells were labeled with annexin V-FITC for 15 min in dark and analyzed by a flow cytometer using 10,000 cells for each measurement. As dead cells lose their membrane integrity, forward and side scatter values (FS/SS in dot plot) were used to exclude debris and dead cells. For the measurement and analysis of data CellQuest Pro software was used. For the numerical comparison, the detected values (FL1 detector) of the annexin V-labeled, viable cells were adjusted with non-labeled samples and the percentage of the annexin positive cells in the treated groups was compared to the percentage of the annexin positive control cells.

The apoptosis assay, carried out by flow cytometry, provided the opportunity to determine the cellular uptake of the anthraquinones having fluorescence property. The intracellular fluorescence intensity of the model cells treated with the compounds could be quantified by FL2 detector of FACSCalibur flow cytometer and the values were compared to autofluorescence of the non-treated controls.

#### Cell adhesion assay

The effect of the anthraquinones and the aqueous extract on cell adhesion of the model cells were assessed using the xCELLigence SP System (Roche Applied Science, Indianapolis, IN, USA). This system allows monitoring the cellular events by measuring electrical impedance in real time manner across gold microelectrodes integrated on the bottom of tissue culture plate, so called E-plate. The detected impedance change depends on the number or the spreading of cells attached to the surface of the electrodes. The change in impedance is represented as Cell Index (CI). The CI is a relative and dimensionless value, and calculated by the following formula:

2 $$ {\text{CI}} = \left( {{\text{Z}}_{\text{i}} - {\text{Z}}_{0} } \right)/{\text{F}}_{\text{i}} $$

where Z_i_ is the impedance at an individual point of time during the experiment, Z_0_ is the impedance at the start of the experiment, and F_i_ is a constant depending on the frequency (F_10kHz_ = 15).

The impedimetric measurement was performed as previously reported [44]. The solutions of the anthraquinones were added in the concentration range of 10^−8^–10^−5^ M and 10,000 cells per well were seeded. The adhesion of our model cells was monitored in every 20 s for at least 12 h at 10 kHz.

The attachment and the spreading of our model cells was characterized by a time course study of the Delta CI values. The Delta CI refers to the difference of CI value at time point of cell inoculation and CI value at a given time point. The adhesion ability of the cells was described by the slope of Delta CI calculated for a 3 h time interval. The slope values were expressed in percentage of the control. The Delta CI and slope values were calculated by the integrated software (RTCA 1.2, Roche Applied Science, Indianapolis, IN, USA). Each data represents the mathematical average of three parallels.

#### Chemotaxis assay

Chemotactic responsiveness of the three cell lines was measured in a NeuroProbe^®^ MBB 96 chamber (NeuroProbe, Gaithensburg, MD, USA) by using polycarbonate filters with pore size 8 μm. The substances were diluted in FCS-free cell culture medium to 10^−8^–10^−5^ M concentrations, except the aqueous extract, for which the final concentration range was 10^−8^–10^−6^ M. The incubation time was 24 h at 37 °C in a humidified 5 % CO_2_ atmosphere. The number of the positive chemotactic responder cells was determined by MTT assay as described above. Identical points of the concentration course study represent average of 8 parallel measurements. The evaluated value was normalized to the control and this value is given as ‘Chemotaxis index’ (Chtx. ind.), in percent.

3 $$ {\text{Chtx}}.{\text{ ind }}\left[ \% \right] = \left( {{{(\overline{OD}_{540,A} - \overline{OD}_{620,A} )} \mathord{\left/ {\vphantom {{(\overline{OD}_{540,A} - \overline{OD}_{620,A} )} {(\overline{OD}_{540,Ctrl} - \overline{OD}_{620,Ctrl} )}}} \right. \kern-0pt} {(\overline{OD}_{540,Ctrl} - \overline{OD}_{620,Ctrl} )}}} \right) \times 100 $$

#### Holographic microscopy

The HoloMonitor™ M4 (Phase Holographic Imaging, Lund, Sweden) was used as an incubator proof, time-lapse and cell tracking instrument for adherent cells. In this equipment development of image applies a non-invasive technique—digital holography—that does not require any labeling or staining. The applied technique is based on how the target cells shift the phase light that passes through the cell. The image is reconstructed on the basis how the cells interfere with the light. The provided data characterize locomotor behavior of the cells as well as describe in vivo morphological alterations elicited by other cells or environmental factors. HoloStudio™ M4 2.5 was used as dedicated software to capture and analyze series of images gained in time-lapse recording of cells.

Special considerations and limits arising from the special technical features of holographic microscope in the present experiment: (1) as laser beam is applied to generate interference patterns we had to limit the tested compounds to alizarin and purpurin as phototoxicity of some components of the aqueous extract is conceivable [26]; (2) development of holographic image is rather difficult and results poor image in some tenuous cell types (e.g. endothelium, fibroblast cells)—therefore in this part of the study only the two melanoma cell lines (A2058 and HT168-M1) were investigated but not MRC-5 cells.

##### Chemokinetic effects

In the present experiments the chemokinetic effects were evaluated by holographic tracking, measurement and calculation of the following parameters: (1) migration—the shortest direct distance from the starting point to the end point (μm); (2) motility (μm)—the actual way traveled from the starting point to the end point (μm); (3) migration directness—ratio of migration and motility (dimensionless); (4) motility speed—the actual way traveled from the starting point to the end point given per hour (μm/h).

The procedure of measurement in brief:

The cells (2.5 × 10^5^) were seeded on a Petri dish (diameter: 35 mm) and cultured for 24 h prior the treatment with compounds in 10^−5^ M final concentration. Following automatic and manual calibrations the proper field of the slide with cells was focused by autofocusing. In time-lapse tracking the settings were: total time = 2 h; interval = 30 s. For evaluation of images of time-lapse automatic background thresholding method was used with “Minimum error sets” algorithm (adjustment = 128) using the minimum error histogram-based threshold method; in object definition, the minimum object size was 36. The number of evaluated cells/image: 50; the total number of evaluated images: 240. For manual cell identification, objects of the marginal zone were abandoned. Reliability of tracking was controlled according to human judgment frame-by-frame.

##### Morphometry

Once the cell movement has been tracked, the cell morphology can also be followed over time by using the Plot Features tab. Different morphological parameters can be displayed. The list below contains all parameters calculated to characterize morphological changes of our tumor model cells (A2058 and HT168-M1). (1) Area—it is the surface area of the image that is occupied by the cell (μm^2^). The calculations are based on a threshold setting that distinguishes background from cell. (2) Thickness avg.—it is the average thickness of the cell (μm). The calculations are based on the phase shift, the wavelength of the light and the refractive index of the cell. (3) Volume— is the optical cell volume (μm^3^), it is based on values of ‘Area’ and ‘Thickness’. (4) Roughness—it is calculated by subtracting a mathematically smoothed image from the actual image in each pixel, and gives an indication of the smoothness/roughness of the cell. The calculations are based on the phase shift in each pixel of the thresholded cell. A healthy cell has usually a low degree of roughness while a dying or dead cell usually has more roughness. (5) Eccentricity—it is how elongated the cell is, or how much the cell deviates from being a circle. A value of 0 corresponds to a circle and the more elongated the cell is the higher the eccentricity value becomes, approaching 1. The calculations are based on a threshold setting that distinguishes background from cell. (6) Hull convexity—it is a measure on how much the 3D cell shape deviates from the perfect convex shape. A cell is usually rather smooth in 3D, a higher value means less dips in cell thickness, i.e. a more perfect shape. (7) Irregularity—it is a measure of how much the circumference of the cell deviates from the circumference of a perfect circle. A value of 0 means the cell is circular and higher values mean a longer, more irregular outline. The calculations are based on a threshold setting that distinguishes background from cell [45].

### Statistical evaluation of data

Data obtained from each experiment represent averages ± S.D. values and were expressed as a percent of the control. Statistical analysis was performed using the one-way ANOVA algorithm of Origin Pro 8.0 (OriginLab Corporation, Northampton, MA, USA) program. In holographic microscopy HoloStudio™ M4 2.5 program was used to analyze data.

Significance levels correspond to *p < 0.05; **p < 0.01; ***p < 0.001.

## Additional files

10.1186/s12935-015-0271-4 Growth inhibitory effect of 48 h long treatment of alizarin, purpurin and aqueous extract in melanoma cell lines (A2058, HT168-M1) and normal fibroblast (MRC-5). **Figure S2**. Time course study of cell adhesion behaviors of the investigated model cells. **Figure S3**. Time course study of cell growth of the melanoma cells. **Figure S4**. Cellular uptake of the anthraquinones (10^−5^ M) by melanoma cell lines (A2058, HT168-M1) and normal fibroblast (MRC-5) after 72 h incubation.
10.1186/s12935-015-0271-4 Holographic morphometry (simple parameters: area, thickness, volume) of A2058 melanoma cells treated with alizarin and purpurin. **Table S2**. Holographic morphometry (complex derived parameters: roughness, eccentricity, Hull convexity, irregularity) of A2058 melanoma cells treated with alizarin and purpurin. **Table S3**. Holographic morphometry (simple parameters: area, thickness, volume) of HT168-M1 melanoma cells treated with alizarin and purpurin. **Table S4**. Holographic morphometry (complex derived parameters: roughness, eccentricity, Hull convexity, irregularity) of HT168-M1 melanoma cells treated with alizarin and purpurin.

## Abbreviations

Chtx. ind.: Chemotaxis index
CI: Cell index
Inh. ind.: Inhibition index
LTB_4_: Leukotriene B4
