# Supplementary material for: Targeted tumor therapy by Rubia tinctorum L.: analytical characterization of hydroxyanthraquinones and investigation of their selective cytotoxic, adhesion and migration modulator effects on melanoma cell lines (A2058 and HT168-M1)
Source: Cancer Cell Int. 2015 Dec 18;15:119. doi: 10.1186/s12935-015-0271-4 (PMC4683936; doi:10.1186/s12935-015-0271-4)
Supplement: Supplementary file 1 — 10.1186/s12935-015-0271-4 Growth inhibitory effect of 48 h long treatment of alizarin, purpurin and aqueous extract in melanoma cell lines (A2058, HT168-M1) and normal fibroblast (MRC-5). Figure S2. Time course study of cell adhesion behaviors of the investigated model cells. Figure S3. Time course study of cell growth of the melanoma cells. Figure S4. Cellular uptake of the anthraquinones (10−5 M) by melanoma cell lines (A2058, HT168-M1) and normal fibroblast (MRC-5) after 72 h incubation. [file 12935_2015_271_MOESM1_ESM.docx]

**Additional figures**

**for**

**Targeted tumor therapy by *Rubia tinctorum* L.: analytical characterization of hydroxyanthraquinones and investigation of their selective cytotoxic, adhesion and migration modulator effects on melanoma cell lines (A2058 and HT168-M1).**

**Eszter Lajkó^1,#^**

Email: [lajesz@gmail.com](mailto:lajesz@gmail.com)

**Péter Bányai^2,#^**

Email: [banyai.peter@pharma.semmelweis-univ.hu](mailto:banyai.peter@pharma.semmelweis-univ.hu)

**Zsófia Zámbó^1^**

Email: [zambozsofia14@gmail.com](mailto:zambozsofia14@gmail.com)

**László Kursinszki^2^**

Email: [kursinszki.laszlo@pharma.semmelweis-univ.hu](mailto:kursinszki.laszlo@pharma.semmelweis-univ.hu)

**Éva Szőke^2^**

Email: [szoke.eva@pharma.semmelweis-univ.hu](mailto:szoke.eva@pharma.semmelweis-univ.hu)

**László Kőhidai^1,^***

*Corresponding author

Email: [kohlasz2@gmail.com](mailto:kohlasz2@gmail.com)

Telephone number: +36-1-210-2930/56232

Fax number: +36-1-303-6968

^1^Department of Genetics, Cell- and Immunobiology, Semmelweis University, Nagyvárad tér 4, Budapest H-1089, Hungary

^2^Department of Pharmacognosy, Semmelweis University, Üllői út 26, Budapest H-1085, Hungary

^#^Lajkó, E. and Bányai, P. are co-first authors, they contributed equally to this work.

**Keywords**

*Rubia tinctorum* L., hydroxyanthraquinone, purpurin, melanoma, targeted therapy, HPLC-MS/MS, cell adhesion, migration, impedimetry, holographic microscope

**Additional figures provide the following results:**

1. Growth inhibitory effect of 48 h long treatment of alizarin purpurin and aqueous extract in melanoma cell lines (A2058, HT168-M1) and normal fibroblast (MRC-5). (Additional file 1: Figure S1)
2. Time course study of cell adhesion behaviors of the investigated model cells. (Additional file 1: Figure S2)
3. Time course study of cell growth of the melanoma cells. (Additional file 1: Figure S3)
4. Cellular uptake of the anthraquinones (10^-5^ M) by melanoma cell lines (A2058, HT168-M1) and normal fibroblast (MRC-5) after 72 h incubation. (Additional file 1: Figure S4)





**Figure S1** Growth inhibitory effect of 48 h long treatment of **(a)** alizarin, **(b)** purpurin and **(c)** aqueous extract in melanoma cell lines (A2058, HT168-M1) and normal fibroblast (MRC-5).

The ‘Inhibition index’ (Inh. ind.) is expressed as a percentage of the control. Data shown in the table represent mathematical averages of six parallels and ±S.D. values. The level of significance is shown as follows: *: p<0.05; **: p<0.01; ***: p<0.001.

**Figure S2** Time course study of cell adhesion behaviors of the model cells.

The Delta Cell index (Delta CI) refers to the difference of CI value at time point of cell inoculation and CI value at a given time point. The Delta CI values in each time point represent mathematical averages of three parallels and ±S.D. values.





**Figure S3** Time course study of cell growth of the melanoma cells.

The Delta Cell index (Delta CI) refers to the difference of CI value at time point of cell inoculation and CI value at a given time point. The Delta CI values in each time point represent mathematical averages of three parallels and ±S.D. values.

Based on the growth curves of A2058 and HT168-M1 cells, the doubling times were calculated by RTCA 1.2 software of the xCELLigence SP.


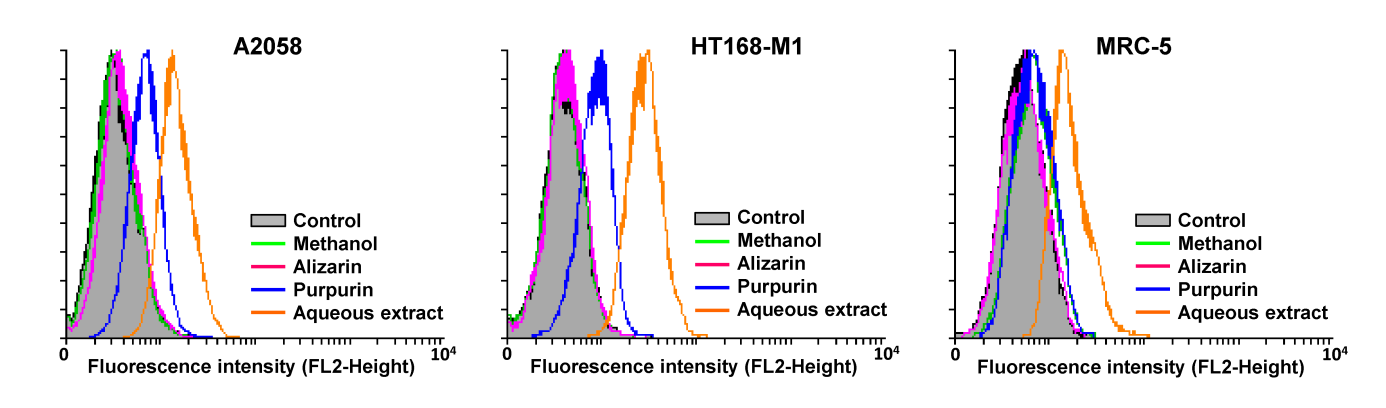


**Figure S4** Cellular uptake of the anthraquinones (10^-5^ M) by melanoma cell lines (A2058, HT168-M1) and normal fibroblast (MRC-5) after 72 h incubation.
