# Supplementary material for: Targeted tumor therapy by Rubia tinctorum L.: analytical characterization of hydroxyanthraquinones and investigation of their selective cytotoxic, adhesion and migration modulator effects on melanoma cell lines (A2058 and HT168-M1)
Source: Cancer Cell Int. 2015 Dec 18;15:119. doi: 10.1186/s12935-015-0271-4 (PMC4683936; doi:10.1186/s12935-015-0271-4)
Supplement: Supplementary file 2 — 10.1186/s12935-015-0271-4 Holographic morphometry (simple parameters: area, thickness, volume) of A2058 melanoma cells treated with alizarin and purpurin. Table S2. Holographic morphometry (complex derived parameters: roughness, eccentricity, Hull convexity, irregularity) of A2058 melanoma cells treated with alizarin and purpurin. Table S3. Holographic morphometry (simple parameters: area, thickness, volume) of HT168-M1 melanoma cells treated with alizarin and purpurin. Table S4. Holographic morphometry (complex derived parameters: roughness, eccentricity, Hull convexity, irregularity) of HT168-M1 melanoma cells treated with alizarin and purpurin. [file 12935_2015_271_MOESM2_ESM.docx]

**Additional tables**

**for**

**Targeted tumor therapy by *Rubia tinctorum* L.: analytical characterization of hydroxyanthraquinones and investigation of their selective cytotoxic, adhesion and migration modulator effects on melanoma cell lines (A2058 and HT168-M1).**

**Eszter Lajkó^1,#^**

Email: [lajesz@gmail.com](mailto:lajesz@gmail.com)

**Péter Bányai^2,#^**

Email: [banyai.peter@pharma.semmelweis-univ.hu](mailto:banyai.peter@pharma.semmelweis-univ.hu)

**Zsófia Zámbó^1^**

Email: [zambozsofia14@gmail.com](mailto:zambozsofia14@gmail.com)

**László Kursinszki^2^**

Email: [kursinszki.laszlo@pharma.semmelweis-univ.hu](mailto:kursinszki.laszlo@pharma.semmelweis-univ.hu)

**Éva Szőke^2^**

Email: [szoke.eva@pharma.semmelweis-univ.hu](mailto:szoke.eva@pharma.semmelweis-univ.hu)

**László Kőhidai^1,^***

*Corresponding author

Email: [kohlasz2@gmail.com](mailto:kohlasz2@gmail.com)

Telephone number: +36-1-210-2930/56232

Fax number: +36-1-303-6968

^1^Department of Genetics, Cell- and Immunobiology, Semmelweis University, Nagyvárad tér 4, Budapest H-1089, Hungary

^2^Department of Pharmacognosy, Semmelweis University, Üllői út 26, Budapest H-1085, Hungary

^#^Lajkó, E. and Bányai, P. are co-first authors, they contributed equally to this work.

**Keywords**

*Rubia tinctorum* L., hydroxyanthraquinone, purpurin, melanoma, targeted therapy, HPLC-MS/MS, cell adhesion, migration, impedimetry, holographic microscope

**Additional tables provide the following results:**

1. Holographic morphometry (simple parameters: area, thickness, volume) of A2058 melanoma cells treated with alizarin and purpurin. (Additional file 2: Table S1)
2. Holographic morphometry (complex derived parameters: roughness, eccentricity, Hull convexity, irregularity) of A2058 melanoma cells treated with alizarin and purpurin. (Additional file 2: Table S2)
3. Holographic morphometry (simple parameters: area, thickness, volume) of HT168-M1 melanoma cells treated with alizarin and purpurin. (Additional file 2: Table S3)
4. Holographic morphometry (complex derived parameters: roughness, eccentricity, Hull convexity, irregularity) of HT168-M1 melanoma cells treated with alizarin and purpurin. (Additional file 2: Table S4)

**Table S1** Holographic morphometry (simple parameters: area, thickness, volume) of A2058 melanoma cells treated with alizarin and purpurin.

| **A2058** | **Contr.**  **Methanol** | **Alizarin**  **(10^-5^ M)** | **Purpurin**  **(10^-5^ M)** |
| --- | --- | --- | --- |
| ***Avg. Area (µm)*** | | | |
| **Mean** | 428.379 ± 0.77 | 691.871 ± 3.35*** | 494.822 ± 2.11 |
| **Slope** | -0.029 ± 0.01 | 0.544 ± 0.03*** | 0.353 ± 0.02*** |
| **R** | 0.169 | 0.729 | 0.748 |
| **Adj. R-Square** | 0.529 | 0.558 | 0.006 |
| ***Avg. Thickness (µm)*** | | | |
| **Mean** | 2.691 ± 0.01 | 2.715 ± 0.01 | 2.238 ± 0.01 |
| **Slope** | -1.17E-04 ± 7.48E-05 | -8.59E-04 ± 6.93E-05 | -0.001 ± 7.64E-05 |
| **R** | 0.101 | 0.625 | 0.691 |
| **Adj. R-Square** | 0.388 | 0.475 | 0.042 |
| ***Avg. Volume (µm^3^)*** | | | |
| **Mean** | 1088.255 ± 2.82 | 1882.982 ± 7.77** | 1053.208 ± 3.52 |
| **Slope** | -0.136 ± 0.04 | 0.916 ± 0.09*** | 0.163 ± 0.05 |
| **R** | 0.216 | 0.529 | 0.207 |
| **Adj. R-Square** | 0.042 | 0.277 | 0.039 |

Data represent variables and ±S.D. values calculated for 50 cell / group in 240 consecutive frames.

The slope values are expressed as dimensionless values to describe the changing rate of Mean values in the 2 hours’ time interval of treatments.

R and Adj. R-Square represent data of linear regression analysis to describe trends of variables belonging to the parameters calculated.

The presented data were calculated by HoloStudio^TM^ M4 2.5 and analyzed Origin Pro 8.0.

The level of significance is shown as follows: *: p<0.05; **: p<0.01; ***: p<0.001.

**Table S2** Holographic morphometry (complex derived parameters: roughness, eccentricity, Hull convexity, irregularity) of A2058 melanoma cells treated with alizarin and purpurin.

| **A2058** | **Contr.**  **Methanol** | **Alizarin**  **(10^-5^ M)** | **Purpurin**  **(10^-5^ M)** |
| --- | --- | --- | --- |
| ***Avg. Roughness*** | | | |
| **Mean** | 5.094 ± 0.02 | 4.375 ± 0.03 | 4.846 ± 0.02 |
| **Slope** | -0.002 ± 3.43E-04 | 0.004 ± 3.77E-04 | -0.001 ± 3.47E-04 |
| **R** | 0.439 | 0.554 | 0.210 |
| **Adj. R-Square** | 0.189 | 0.304 | 0.040 |
| ***Avg. Eccentricity*** | | | |
| **Mean** | 0.738 ± 0.01 | 0.683 ± 0.01 | 0.753 ± 0.01 |
| **Slope** | 2.38E-4 ± 2.18E-5 | -8.08E-5 ± 2.35E-5*** | 1.38E-4 ± 2.11E-5 |
| **R** | 0.577 | 0.216 | 0.391 |
| **Adj. R-Square** | 0.331 | 0.042** | 0.150* |
| ***Avg. Hull convexity*** | | | |
| **Mean** | 0.960 ± 2.69E-4 | 0.948 ± 4.87E-4 | 0.944 ± 3.49E-4 |
| **Slope** | -1.30E-5 ± 3.80E-6 | -6.60E-5 ± 5.57E-6** | -3.63E-5 ± 4.47E-6* |
| **R** | 0.217 | 0.607 | 0.465 |
| **Adj. R-Square** | 0.043 | 0.366 | 0.213 |
| ***Avg. Irregularity*** | | | |
| **Mean** | 0.284 ± 0.01 | 0.328 ± 0.01 | 0.341 ± 0.01* |
| **Slope** | 2.17E-4 ± 1.75E-5 | 3.04E-4 ± 1.66E-5 | 3.29E-4 ± 2.40E-5 |
| **R** | 0.625 | 0.764 | 0.665 |
| **Adj. R-Square** | 0.389 | 0.582 | 0.440 |

Data represent variables and ±S.D. values calculated for 50 cell / group in 240 consecutive frames.

The slope values are expressed as dimensionless values to describe the changing rate of Mean values in the 2 hours’ time interval of treatments.

R and Adj. R-Square represent data of linear regression analysis to describe trends of variables belonging to the parameters calculated.

The presented data were calculated by HoloStudio^TM^ M4 2.5 and analyzed Origin Pro 8.0.

The level of significance is shown as follows: *: p<0.05; **: p<0.01; ***: p<0.001.

**Table S3** Holographic morphometry (simple parameters: area, thickness, volume) of HT168-M1 melanoma cells treated with alizarin and purpurin.

| **HT168-M1** | **Contr.**  **Methanol** | **Alizarin**  **(10^-5^ M)** | **Purpurin**  **(10^-5^ M)** |
| --- | --- | --- | --- |
| ***Avg. Roughness*** | | | |
| **Mean** | 3.264 ± 0.04 | 3.400 ± 0.04 | 2.830 ± 0.03 |
| **Slope** | 0.002 ± 6.80E-4 | -0.008 ± 2.70E-4 | -3.85E-5 ± 4.17E-4*** |
| **R** | 0.205 | 0.882 | 0.005 |
| **Adj. R-Square** | 0.038 | 0.778 | -0.004 |
| ***Avg. Eccentricity*** | | | |
| **Mean** | 0.682 ± 0.01 | 0.671 ± 0.01 | 0.710 ± 0.01 |
| **Slope** | 6.55E-5 ± 2.26E-5 | -7.12E-5 ± 1.90E-5 | 7.31E-5 ± 1.46E-5 |
| **R** | 0.184 | 0.235 | 0.307 |
| **Adj. R-Square** | 0.030 | 0.051 | 0.090 |
| ***Avg. Hull convexity*** | | | |
| **Mean** | 0.944 ± 3.85E-4 | 0.950 ± 5.13E-4 | 0.965 ± 3.86E-4 |
| **Slope** | -2.91E-5 ± 5.24E-6 | -1.48E-5 ± 7.33E-6 | -3.22E-5 ± 5.15E-6 |
| **R** | 0.339 | 0.129 | 0.375 |
| **Adj. R-Square** | 0.111 | 0.012* | 0.137 |
| ***Avg. Irregularity*** | | | |
| **Mean** | 0.396 ± 0.01 | 0.401 ± 0.01 | 0.373 ± 0.01 |
| **Slope** | 2.77E-4 ± 2.13E-5 | 3.91E-4 ± 1.65E-5 | 1.84E-4 ± 1.22E-5 |
| **R** | 0.643 | 0.838 | 0.698 |
| **Adj. R-Square** | 0.412 | 0.701 | 0.486 |

Data represent variables and ±S.D. values calculated for 50 cell / group in 240 consecutive frames.

The slope values are expressed as dimensionless values to describe the changing rate of Mean values in the 2 hours’ time interval of treatments.

R and Adj. R-Square represent data of linear regression analysis to describe trends of variables belonging to the parameters calculated.

The presented data were calculated by HoloStudio^TM^ M4 2.5 and analyzed Origin Pro 8.0.

The level of significance is shown as follows: *: p<0.05; **: p<0.01; ***: p<0.001.

**Table S4** Holographic morphometry (complex derived parameters: roughness, eccentricity, Hull convexity, irregularity) of HT168-M1 melanoma cells treated with alizarin and purpurin.

| **HT168-M1** | **Contr.**  **Methanol** | **Alizarin**  **(10^-5^ M)** | **Purpurin**  **(10^-5^ M)** |
| --- | --- | --- | --- |
| ***Avg. Roughness*** | | | |
| **Mean** | 3.264 ± 0.04 | 3.400 ± 0.04 | 2.830 ± 0.03 |
| **Slope** | 0.002 ± 6.80E-4 | -0.008 ± 2.70E-4 | -3.85E-5 ± 4.17E-4*** |
| **R** | 0.205 | 0.882 | 0.005 |
| **Adj. R-Square** | 0.038 | 0.778 | -0.004 |
| ***Avg. Eccentricity*** | | | |
| **Mean** | 0.682 ± 0.01 | 0.671 ± 0.01 | 0.710 ± 0.01 |
| **Slope** | 6.55E-5 ± 2.26E-5 | -7.12E-5 ± 1.90E-5 | 7.31E-5 ± 1.46E-5 |
| **R** | 0.184 | 0.235 | 0.307 |
| **Adj. R-Square** | 0.030 | 0.051 | 0.090 |
| ***Avg. Hull convexity*** | | | |
| **Mean** | 0.944 ± 3.85E-4 | 0.950 ± 5.13E-4 | 0.965 ± 3.86E-4 |
| **Slope** | -2.91E-5 ± 5.24E-6 | -1.48E-5 ± 7.33E-6 | -3.22E-5 ± 5.15E-6 |
| **R** | 0.339 | 0.129 | 0.375 |
| **Adj. R-Square** | 0.111 | 0.012* | 0.137 |
| ***Avg. Irregularity*** | | | |
| **Mean** | 0.396 ± 0.01 | 0.401 ± 0.01 | 0.373 ± 0.01 |
| **Slope** | 2.77E-4 ± 2.13E-5 | 3.91E-4 ± 1.65E-5 | 1.84E-4 ± 1.22E-5 |
| **R** | 0.643 | 0.838 | 0.698 |
| **Adj. R-Square** | 0.412 | 0.701 | 0.486 |

Data represent variables and ±S.D. values calculated for 50 cell / group in 240 consecutive frames.

The slope values are expressed as dimensionless values to describe the changing rate of Mean values in the 2 hours’ time interval of treatments.

R and Adj. R-Square represent data of linear regression analysis to describe trends of variables belonging to the parameters calculated.

The presented data were calculated by HoloStudio^TM^ M4 2.5 and analyzed Origin Pro 8.0.

The level of significance is shown as follows: *: p<0.05; **: p<0.01; ***: p<0.001.
